# USP5 facilitates bladder cancer progression by stabilizing the c-Jun protein

**DOI:** 10.1186/s12935-024-03222-7

**Published:** 2024-01-16

**Authors:** Hui-hui Zhang, An-qi Zhang, Peng Peng, Liang Huang, Cai-ying Liu, Xin-rui Nie, De-fu Hou, Xia Zhang, Shang-ze Li

**Affiliations:** 1 Department of Laboratory Medicine, Key Laboratory of Study and Discovery of Small Targeted Molecules of Hunan Province, Hunan Normal University School of Medicine, 371 Tongzipo Road, Yuelu District, Changsha, Hunan China; 2 School of Medicine, Chongqing University, 131 Yubei Road, Shapingba District, Chongqing, China

**Keywords:** USP5, c-Jun, Deubiquitination, Bladder cancer

## Abstract

**Background:**

Bladder cancer is the second most common genitourinary malignancy worldwide. The death rate of bladder cancer has increased every year. However, the molecular mechanism of bladder cancer is not sufficiently studied. Deubiquitinating enzymes (DUBs) play an important role in carcinogenesis. Several studies have demonstrated that USP5 associated with malignancy and pathological progression in hepatocellular carcinoma, colorectal and non-small cell lung cancer. However, the role of USP5 in bladder cancer need to be explored.

**Methods:**

The USP5 expression was analysed using the web server GEPIA. To explore USP5 function in bladder cancer, we constructed USP5-knockout cell lines in T24 cells. A FLAG-USP5 (WT USP5) plasmid and a plasmid FLAG-USP5 C335A (catalytic-inactive mutant) used to overexpress USP5 in EJ cells. CCK8, colony formation, transwell and scratch assays were used to assess cell viability, proliferation and migration. RNA sequencing (RNA-seq) and dual-luciferase reporter assays were performed to screen the pathway. Coimmunoprecipitation and immunofluorescence were used to explore the interaction between USP5 and c-Jun. Cycloheximide (CHX) chase assays were performed to establish the effect of USP5 on c-Jun stability. Xenograft mouse model was used to study the role of USP5 in bladder cancer.

**Results:**

USP5 expression is increased in bladder cancer patients. Genetic ablation of USP5 markedly inhibited bladder cancer cell proliferation, viability, and migration both in vitro and in vivo. RNA-seq and luciferase pathway screening showed that USP5 activated JNK signalling, and we identified the interaction between USP5 and c-Jun. USP5 was found to activate c-Jun by inhibiting its ubiquitination.

**Conclusions:**

Our results show that high USP5 expression promotes bladder cancer progression by stabilizing c-Jun and that USP5 is a potential therapeutic target in bladder cancer.

**Supplementary Information:**

The online version contains supplementary material available at 10.1186/s12935-024-03222-7.

## Introduction

Bladder cancer (BC), a carcinoma of the urothelial system, is one of the most common malignancies worldwide, with over 550,000 patients diagnosed and 190,000 succumbed to the disease worldwide every year [1]. Cigarette smoking, male sex, and advanced age contribute to the development of bladder cancer. Bladder cancer is classified into two subclasses: non-muscle-invasive bladder cancer (NMIBC) and muscle-invasive bladder cancer (MIBC). Tumours restricted to the urothelium and the lamina propria are classified as NMIBC, which is not life-threatening. NMIBCs account for approximately 70% of newly diagnosed BC cases, and the five-year survival rate of NMIBC is above 90% [2]. Tumours that invade the detrusor muscle are classified as MIBC. MIBC is accompanied by invasion and metastasis, which have increased the death rate annually. The five-year survival rate of MIBC is 50% [3]. NMIBCs are treated with endoscopic resection and adjuvant intravesical therapy. For patients with MIBC, radical cystectomy and urinary diversion or trimodal therapy with maximal endoscopic resection, radiosensitizing chemotherapy, and radiation is warranted [4]. The advent of checkpoint inhibitors, targeted therapies, and antibody-drug conjugates for immunotherapy has greatly improved the treatment of bladder cancer. Improved understanding of the molecular biology of bladder cancer is important for the development of diagnosis and treatment.

The c-Jun N-terminal kinase (JNK) pathway is a mitogen-activated protein kinase (MAPK) pathway [5]. JNK signalling is involved in many physiological processes and pathological conditions, including inflammation, neurodegenerative diseases and multiple tumorigenic processes [6–10]. JNK plays pivotal roles in aspects related to bladder cancer, such as tumorigenesis [11, 12], apoptosis [13], the chemotherapy response [14] and metastasis [15]. The transcription factor activator protein 1 (AP-1) may be activated by MAPKs, particularly JNK. c-Jun is a member of the Jun family and is a component of AP-1 complexes [16]. c-Jun was the first purely oncogenic transcription factor discovered. It is important in processes related to cellular homeostasis, including proliferation, apoptosis and survival. The relationship between c-Jun and tumorigenesis has been widely investigated [17, 18]. Usually, c-Jun is activated by JNK, which binds to the c-Jun transactivation domain and phosphorylates it at Ser63 and Ser73 [19]. c-Jun is degraded through a ubiquitination mechanism. Several studies have revealed that c-Jun is ubiquitinated by several E3 ubiquitin ligases [20, 21]. However, the regulation of the c-Jun by deubiquitinating enzymes (DUBs) requires further study.

The ubiquitin‒proteasome system is responsible for protein stability. DUBs remove ubiquitin moieties from substrates. DUBs can be classified into five families based on their sequence and structural homology [22]: Otubain proteases (OTUs), ubiquitin C-terminal hydrolases (UCHs), ubiquitin-specific proteases (USPs), Machado-Joseph disease proteases (MJDs), and JAB1/MPN/Mov34 metalloenzymes (JAMMs). USP5 is a cysteine deubiquitinating enzyme belonging to the USP family. The *USP5* gene is located on chromosome 12p13 and encodes the 93.3-kDa protein USP5 [23]. Analysis of data in the Human Protein Atlas (https://www.proteinatlas.org/) revealed that USP5 is highly expressed in testicular cancer, prostate cancer, breast cancer and urothelial cancer. Several studies have demonstrated that USP5 plays an important role in cancers by targeting its substrates. In hepatocellular carcinoma (HCC) and colorectal cancer (CRC), USP5 is highly expressed and closely associated with malignancy and pathological progression [24]. In pancreatic ductal adenocarcinoma (PDAC), USP5 stabilizes FoxM1 to promote tumour growth [25]. In non-small cell lung cancer, USP5 promotes cell proliferation, colony formation and migration [26]. However, the role of USP5 in BC needs to be explored.

We found that USP5 was overexpressed in bladder cancer samples and that patients with high USP5 expression had an unfavourable prognosis. In vitro, USP5 overexpression promotes the proliferation and migration of EJ bladder cancer cells. Consistent with these results, USP5 deficiency inhibits the proliferation and migration of T24 bladder cancer cells. Through RNA sequencing (RNA-seq) and luciferase assays, we found that USP5 activates the JNK pathway. We showed that USP5 binds to and stabilizes c-Jun by mediating its deubiquitination, thereby promoting the JNK signalling cascade. Finally, we revealed a novel mechanism of USP5 in bladder cancer development and progression.

## Materials and methods

### Antibodies and plasmids

The following antibodies were used: rabbit polyclonal anti-USP5 (10473-1-AP, Proteintech, Wuhan, China); rabbit monoclonal anti-c-Jun (ab40766, Abcam, Waltham, USA, 1:2000 dilution); mouse anti-HA (M180-3, MBL, Japan, 1:5000 dilution); mouse anti-Flag (M185-11R, MBL, Japan, 1:5000 dilution); mouse anti-Myc (M192-3, MBL, Japan,1:5000 dilution); mouse anti-GAPDH (ANT011, AntGene, Wuhan, China, 1:5000 dilution); HRP-labelled goat anti-mouse IgG (H + L) (A0216, Beyotime, Shanghai, China, 1:5000 dilution); and HRP goat anti-rabbit IgG (H + L) (ANT020, AntGene, Wuhan, China, 1:5000 dilution).

The plasmids PHAGE-3×Flag-USP5(FLAG-USP5), pHAGE-3×HA-USP5 and pHAGE-3×HA-USP5 C335A (HA-USP5 C335A) were constructed according to the methods in the “Molecular Cloning Experiment Guide”. The plasmid pcDNA3.1-3xFlag-c-Jun (FLAG-c-Jun) was purchased from Youbio (F118284).

### Xenografts

USP5^−/−^ and parental T24 cells were collected and washed twice with PBS. A total of 5 × 10^6^ cells were resuspended in 0.2 mL of PBS and inoculated into the flanks of 5 4-week-old female BALB/c nude mice. Tumours were measured every other day after the appearance of subcutaneous tumours. The tumour volume was calculated as follows: volume = (length×width^2^) × 0.5. 30 days after inoculation, the mice will be deeply anesthetized with isoflurane (5%) for approximately 3 min. Then, the mice were killed by cervical dislocation. All animal studies were conducted in accordance with the Guidelines of the China Animal Welfare Legislation and were approved by the Committee on Ethics in the Care and Use of Laboratory Animals of Hunan Normal University (permit number: 2,021,286). All efforts were made to minimize animal suffering.

### Cell culture and cell lines

All cell lines were purchased from ATCC. HEK293T cells were cultured in Dulbecco’s modified Eagle’s medium (DMEM, HyClone, Logan, USA). T24 cells were maintained in McCoy’s 5a medium (BasalMedia, Shanghai, China). EJ cells were maintained in RPMI 1640 medium (Gibco, Carlsbad, USA). The media were supplemented with 10% foetal bovine serum (FBS, Gibco, Carlsbad, USA) and 1% penicillin/streptomycin (Gibco, Carlsbad, USA). All cells were cultured at 37 °C in a 5% CO_2_ incubator. No mycoplasma contamination was detected.

### Cell proliferation, colony formation, transwell and scratch assays

A Cell Counting Kit-8 (CCK8, BS350B, Biosharp, China) was used for cell proliferation assays. A total of 1 × 10^3^ cells/well were seeded into 96-well plates. CCK8 solution (10 µL in 100 µL medium) was added to each well and incubated at 37 °C for 1 h. The optical density was measured at a wavelength of 450 nm. Colony formation was performed as we previously described. Briefly, 4 × 10^2^ cells were cultured in 6-well plates for 14 days. Colonies were stained with 0.025% crystal violet, and images were acquired using a scanner. For transwell assays, medium with 40% FBS was added to the lower chamber, and serum-free medium was added to the upper chamber. Cells were seeded into the upper chamber. After 36–48 h, cells that migrated into the lower chamber were stained with 0.02% crystal violet. For the scratch assays, cells subjected to different treatments were seeded in 6-well plates. When the cells formed a confluent monolayer, a cell-free area was artificially created in the centre of each well. Images of the wounds were acquired every 12 h using a light microscope.

### Western blot analysis

Cells were lysed with SDS lysis buffer (62.5 mM Tris-HCl (pH 6.8), 2% SDS, and 10% glycerol) at 95 °C for 10 min. Total protein was separated by 10% SDS‒PAGE and transferred to PVDF membranes (IPVH00010; Millipore, Billerica, MA, USA). The membranes were blocked with 5% skim milk for 1 h. The membranes were incubated with primary antibodies overnight at 4 °C. The next day, the membranes were washed in TBST and then incubated with HRP-labelled secondary antibodies at room temperature for 1 h. A Tanon 5500 chemiluminescence image analysis system (Tanon, Shanghai, China) was used to evaluate the chemiluminescence of the protein bands. GAPDH was used as the internal control.

### Coimmunoprecipitation

Cells were lysed using NP-40 lysis buffer (20 mM Tris-HCl (pH 7.4), 150 mM NaCl, and 1% NP-40) in the presence of protease inhibitor cocktails. The lysates were centrifuged at 12,000 rpm for 10 min at 4 °C and then incubated with the approprioat antibody and rProtein A/G Magarose Beads (SM005002, SMART Lifesciences, China) overnight at 4 °C. The next day, the magarose beads were washed with lysis buffer 3 times. The immunoprecipitated proteins were boiled in 2 × SDS‒PAGE loading buffer for 10 min at 95 °C and separated using SDS‒PAGE.

### Luciferase assay

HEK293T cells (30% confluence) were seeded into 24-well plates. The reporter plasmid (100 ng, contains 34 signaling pathways), pRL-CMV (5 ng) with the indicated gene-expressing plasmids (Flag-USP5, 500 ng) or empty vector were transient transfected into the HEK293T cells in each well. were transfected into the cells. After 48 h, luciferase reporter assays were performed with a dual luciferase assay kit (E1960, Promega, Beijing).

### Immunofluorescence

Cells were fixed with 4% paraformaldehyde for 15 min, blocked with 0.1% Triton X-100 and then washed with PBS. The samples were then stained with a primary antibody and the corresponding secondary antibody. Finally, the slides were observed and digitally photographed using a confocal microscope (Leica TCS SP8 SR. Leica, Germany).

### Immunohistochemistry and H&E staining

Bladder cancer tissue microarrays were purchased from Wuhan Shuangxuan Biotechnology Co., Ltd. (Cat. No. IWLT-N-140BL61, contains 64 bladder cancer tissues and 48 normal tissues). Immunohistochemistry examination with USP5 antibody at WuHan Servicebio Technology Co., Ltd. Tumors of each mouse were prepared for histopathological sections, and subjected to HE staining at WuHan Servicebio Technology Co., Ltd.

### Obtaining USP5-knockout cell lines using CRISPR-CAS9

USP5-knockout cells were obtained by clustered regularly interspaced short palindromic repeats (CRISPR)/Cas9-mediated genome editing [27]. Briefly, sgRNAs targeting USP5 exon 6 were designed via the CRISPR Design Tool (sgRNA-1: TGTGGGCGACGCTACTTCGA, sgRNA-2: TACCCGTTAGCTGTCAAGCT). The annealed sgRNA oligos were inserted into the lentiCRISPRv2 vector (Addgene plasmid 52,961) [28] to generate the USP5 knockout plasmids. Transfection and infection were performed using PEI MAX transfection reagent (Polysciences) according to the manufacturer’s instructions as previously described [29]. Positive cells were selected by puromycin (1 ug/ml). The western blot analysis was used for cell line identification.

### Statistical analysis

All experiments were performed 3 times. Two-tailed unpaired Student’s t test was used to compare two groups of data. One-way ANOVA was used to compare multiple groups of data. A P value of less than 0.05 was considered significant. KEGG pathway enrichment analysis was performed using the BGI Dr.Tom.

## Results

### USP5 is overexpressed in bladder cancer

**Fig. 1:**
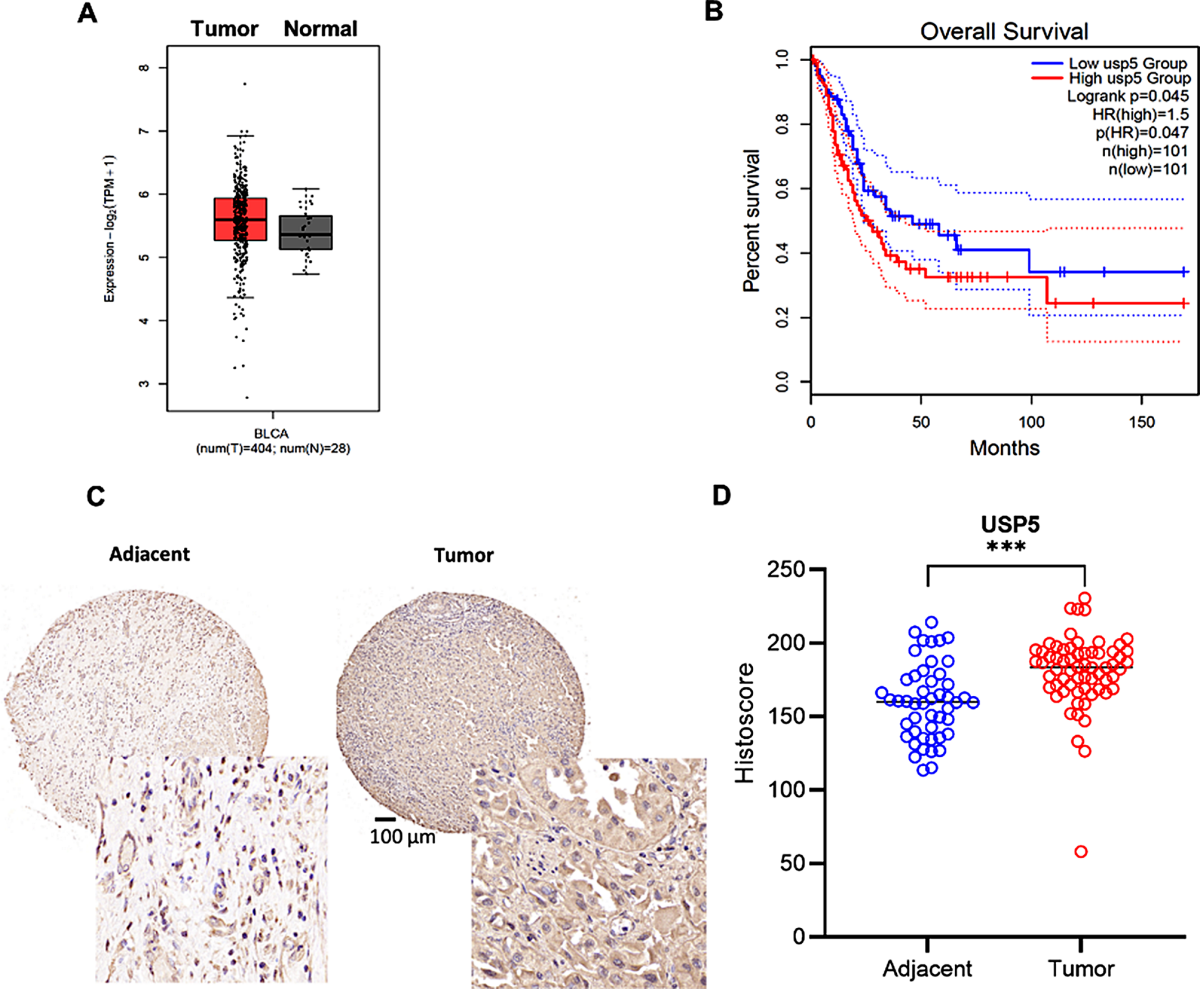
USP5 is overexpressed in bladder cancer. **A** USP5 expression in bladder urothelial carcinoma tissues compared with normal tissues in the GEPIA web server. **B** Analysis of overall survival for 202 patients with bladder cancer based on USP5 expression in the GEPIA web server. The high USP5 group showed significantly lower survival rates (*P* = 0.045). **C** Immunohistochemical staining of USP5 in tissue microarrays containing bladder cancer (×10,×40) and normal tissues (×10,×40). **D** Tissue microarray data analysis of USP5 expression in tumor (*n* = 68) and adjacent normal (*n* = 48) tissues from 68 patients with bladder cancer (P<0.001). Data are presented as the mean ± SEM. Statistical significance was analyzed by Student *t* test

Several studies have shown USP5 overexpression in some types of cancer. We used the web server GEPIA (http://gepia.cancer-pku.cn/) to analyse USP5 expression in bladder cancer. As shown in Fig. 1A, USP5 expression was higher in tumour tissue than in normal tissue. Then, we performed overall survival analysis with data from GEPIA, and the results suggested that bladder cancer patients with high USP5 expression had poorer survival outcomes than those with low USP5 expression (Fig. 1B). To verify USP5 expression in bladder cancer, we analysed USP5 protein levels in bladder cancer tissues and adjacent bladder tissues from patients using immunohistochemistry (IHC). The results suggested that USP5 was obviously highly expressed in bladder cancer compared with normal tissue (Fig. 1C, D). All the results showed that USP5 is closely related to bladder cancer.

### Overexpression of USP5 promotes cell proliferation and migration

**Fig. 2:**
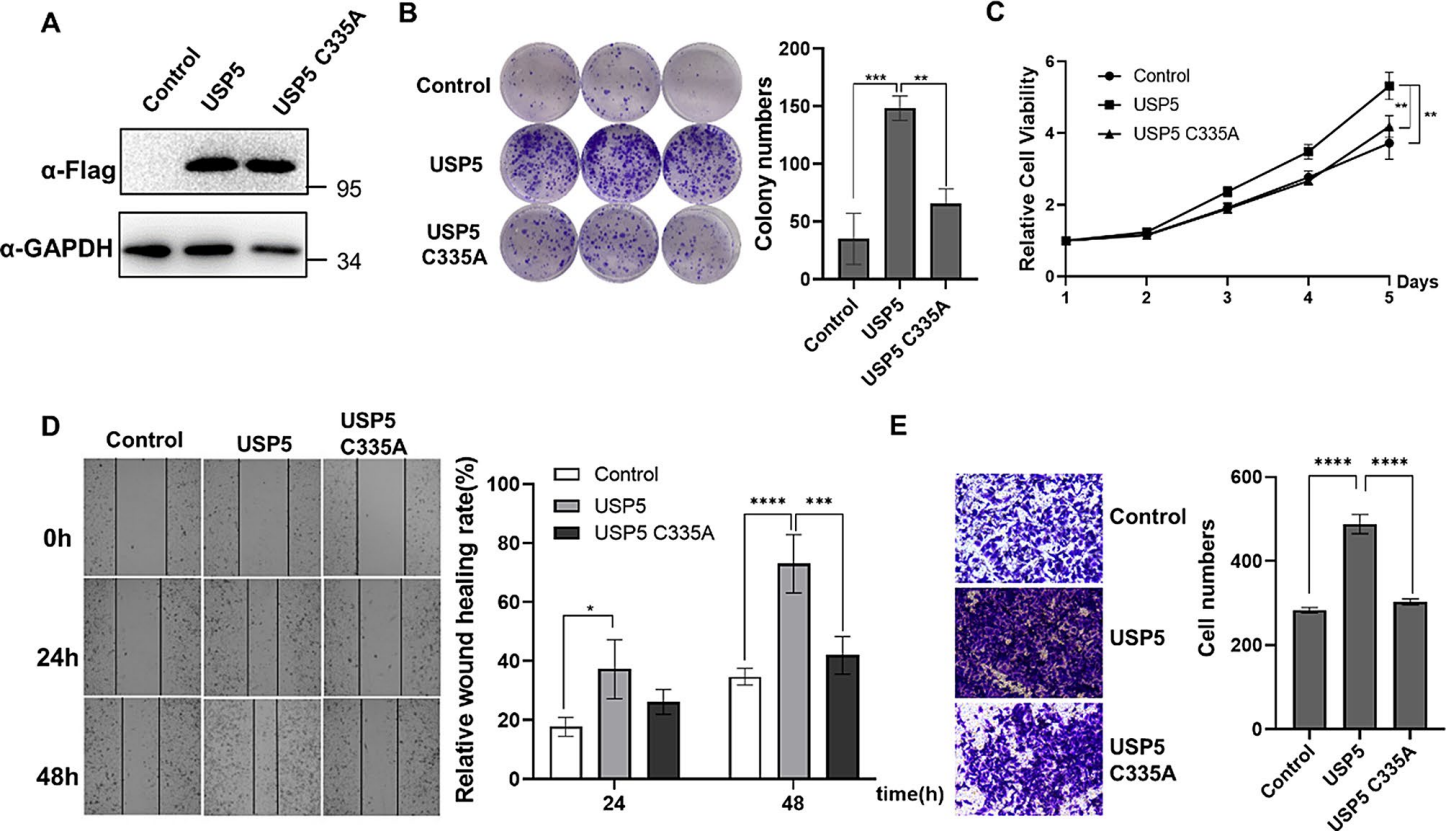
USP5 overexpression promotes bladder cancer cell proliferation, viability and migration. **A** EJ cells were transfected with FLAG-USP5 or FLAG-USP5 C335A as indicated, and protein levels were measured by western blotting. GAPDH served as a control. **B** EJ cells were transfected with USP5 or FLAG-USP5 C335A as indicated, and wild-type EJ cells served as controls. Colony formation assays were performed to evaluate cell viability. The colonies were stained with crystal violet and photographed. The number of colonies was determined and plotted (*n* = 3). **C** EJ cells were transfected with FLAG-USP5 or FLAG-USP5 C335A as indicated, and wild-type EJ cells served as controls. CCK-8 assays were used to analyse cell proliferation (*n* = 6). **D** EJ cells were transfected with FLAG-USP5 or FLAG-USP5 C335A as indicated, and wild-type EJ cells served as controls. Scratch assays were used to evaluate the effects of USP5 overexpression on cell migration and wound repair. The cells were imaged (left, ×20 magnification) and counted, and the results were plotted (right, *n* = 3). The data (mean ± SEM) are representative of 3 independent experiments. **E** EJ cells were transfected with FLAG-USP5 or FLAG-USP5 C335A as indicated, and wild-type EJ cells served as controls. Transwell experiments were used to evaluate the effects of USP5 overexpression on cell migration. The cells were imaged (left, ×20 magnification) and counted, and the results were plotted (right, *n* = 3). The data (mean ± SEM) are representative of 3 independent experiments. Statistical significance was analysed by ANOVA or Student’s t test. *P < 0.05, **P < 0.01, ****P < 0.0001

To clarify the function of USP5 in bladder cancer cells, we examined USP5 expression levels using laboratory-preserved bladder cancer cell lines (UMUC3, T24, EJ) (Fig. S1). A FLAG-USP5 (WT USP5) plasmid and a plasmid expressing a catalytically inactive mutant (FLAG-USP5 C335A) were used to overexpress USP5 in EJ cells with low USP5 expression, and USP5 expression was measured by western blotting (Fig. 2A). We evaluated the effects of USP5 on proliferation and migration using colony formation, cell proliferation, transwell, and scratch assays. The colony formation and cell proliferation assay results showed that exogenous overexpression of USP5 increased colony numbers and cell proliferation, but overexpression of USP5 C335A did not have the same effects (Fig. 2B, C). The results of transwell and scratch assays also showed that overexpression of USP5 but not USP5 C335A significantly enhanced the migration ability of EJ cells (Fig. 2D, E). The above results suggested that USP5 overexpression but not USP5 C335A overexpression promotes bladder cancer cell survival, proliferation and migration.

### USP5 deficiency inhibits cell proliferation and migration

**Fig. 3:**
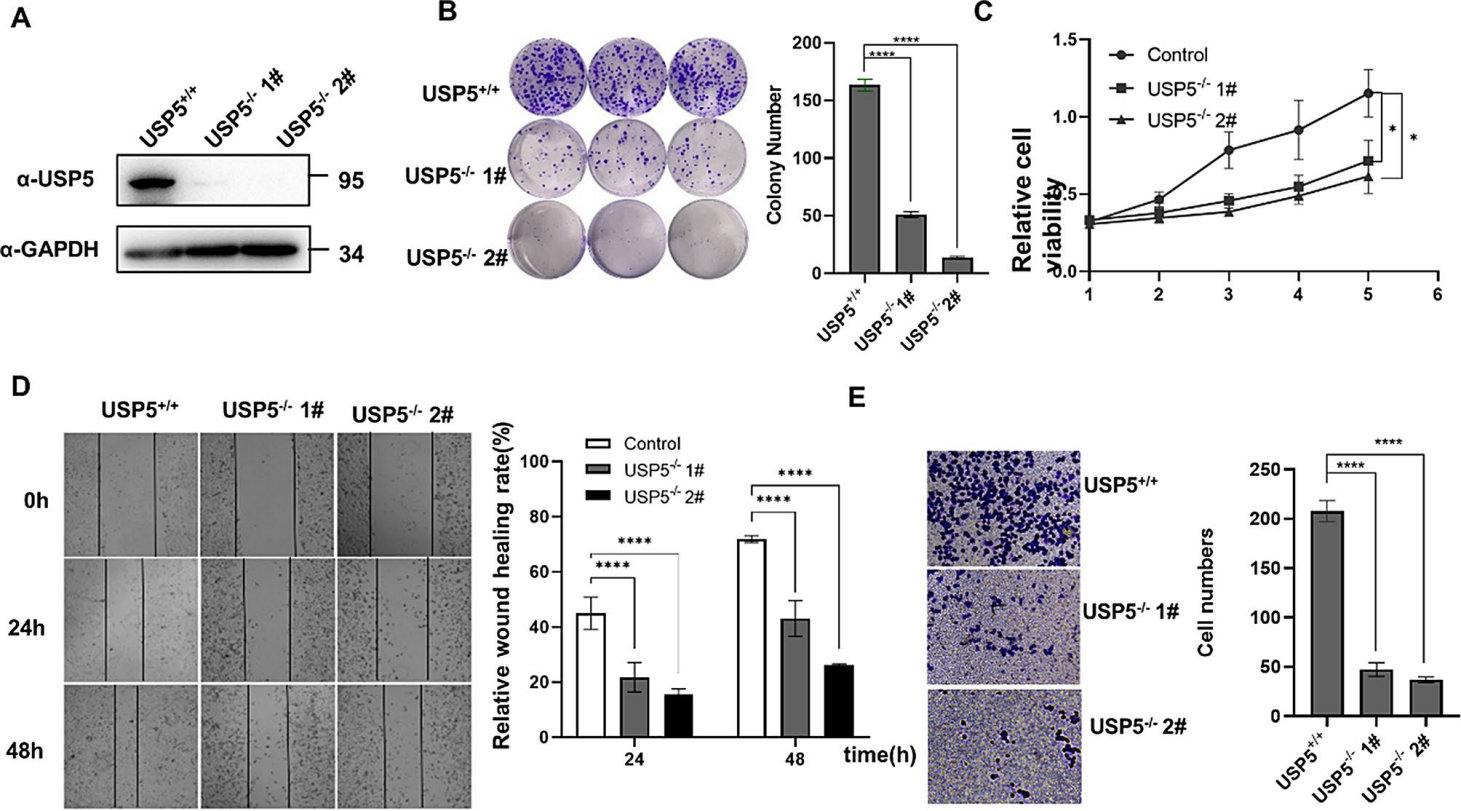
USP5 deficiency inhibits cell proliferation, viability and migration. **A** USP5 protein levels in WT and USP5-deficient T24 cells were measured by western blotting with GAPDH as a loading control. **B** Colony formation assays showed the viability of USP5-deficient T24 bladder cancer cells. Colonies were stained with crystal violet and subsequently imaged (left). The number of colonies was determined and plotted (right, *n* = 3). **C** Cell proliferation was analysed by CCK-8 assays with daily measurements for 5 d (*n* = 6). **D** Scratch assays were used to evaluate the effects of USP5 deficiency on T24 cells. The cells were imaged (left, ×20 magnification) and counted, and the results were plotted (right, *n* = 3). The data (mean ± SEM) are representative of 3 independent experiments. **E** Transwell experiments were used to evaluate the effects of USP5 deficiency on T24 bladder cancer cell migration. The cells were imaged (left, ×20 magnification) and counted, and the results were plotted (right, *n* = 3). The data (mean ± SEM) are representative of 3 independent experiments. Statistical significance was analysed by ANOVA or Student’s t test. *P < 0.05, ****P < 0.0001

For further validation, we constructed USP5-knockout cell lines using the CRISPR-CAS9 technique in T24 cells with high USP5 expression. Western blotting was used to examine the expression of USP5 (Fig. 3A). The results of the colony formation assay revealed that USP5 depletion dramatically decreased the colony formation capability of T24 cells (Fig. 3B). We next tested the effect of USP5 on the proliferation of T24 cells by a CCK8 assay. As shown in Fig. 3C, the proliferation of the knockout cells was substantially slower than that of the wild-type T24 cells. The scratch and transwell assay results also revealed a similar phenotype (Fig. 3D, E). Collectively, these findings indicated that knocking down USP5 markedly inhibited the proliferation, viability, and migration of T24 cells.

### USP5 regulates the JNK pathway

**Fig. 4:**
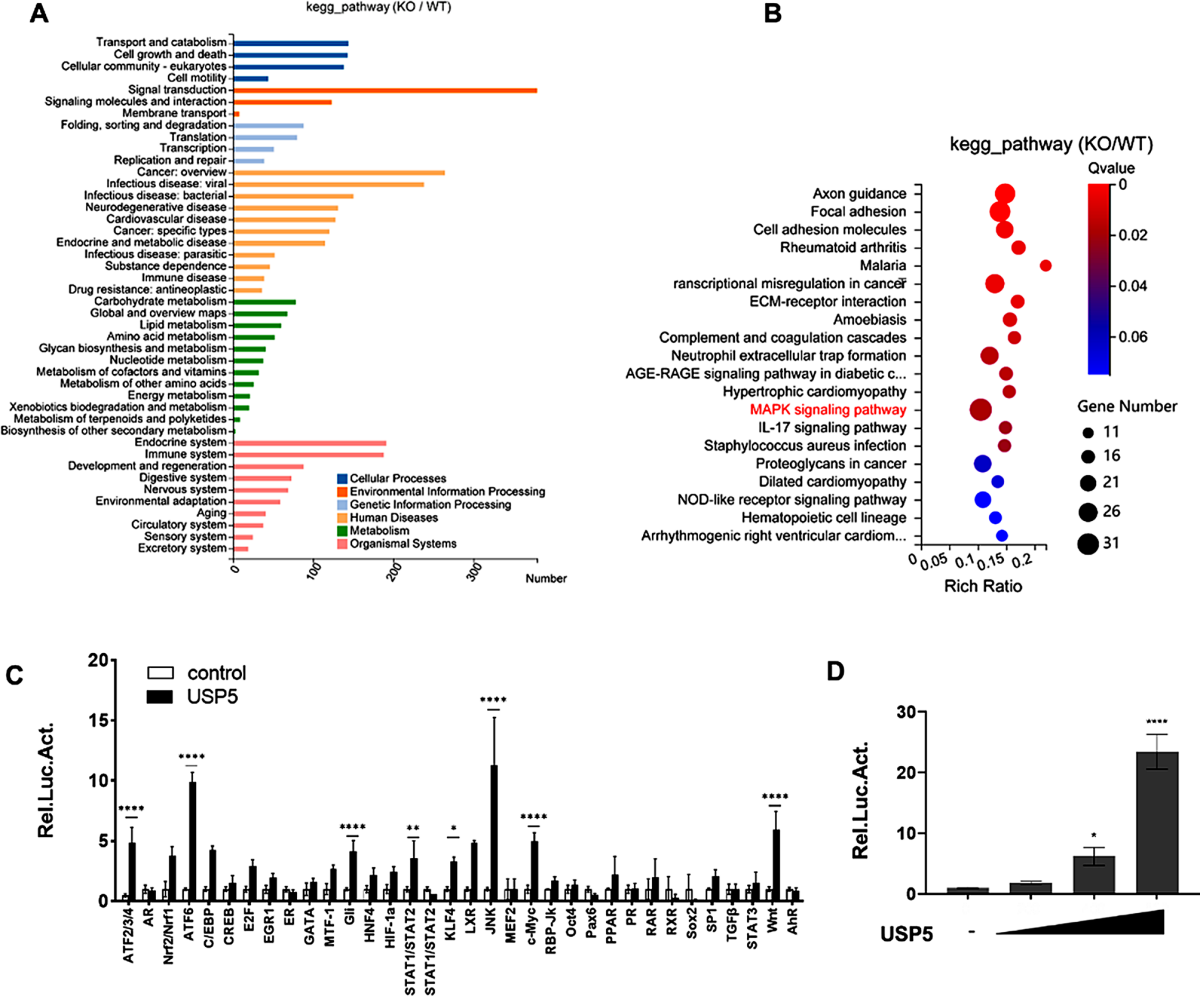
USP5 regulates the JNK pathway. **A** KEGG pathway enrichment analysis on the basis of the most significantly differentially expressed genes between the WT and USP5-deficient groups (*P* < 0.05 using Fisher’s exact test). **B** Bubble diagram showing the enrichment of differentially expressed genes in the biological process category. **C** Luciferase pathway screening revealed that USP5 significantly promoted JNK pathway activation in HEK293T cells (*n* = 3). **D** Luciferase assays showing JNK pathway activity in HEK293T cells transfected with increasing amounts (0, 200, 400, 600 ng) of the USP5 expression plasmid (*n* = 3). The data (mean ± SEM) are representative of 3 independent experiments. Statistical significance was analysed by ANOVA or Student’s t test. *P < 0.05, **P < 0.01, ****P < 0 0.0001

To investigate the molecular mechanism of USP5 in bladder cancer, we performed RNA-seq analysis using USP5-deficient and WT T24 cells. KEGG pathway enrichment analysis was conducted to identify changes in signalling pathways (Fig. 4A). The results revealed that multiple signalling pathways involving numerous biological processes were affected in USP5-deficient cells. The ratio of total genes enriched by MAPK signalling pathway was the largest (Fig. 4B). We also performed luciferase pathway screening using cancer-related reporters in HEK293T cells to identify the underlying pathway. The results showed that USP5 expression noticeably activated the JNK reporter which belongs to the MAPK signalling pathway (Fig. 4C). Furthermore, JNK reporter activity was activated by USP5 in a dose-dependent manner (Fig. 4D).

### USP5 interacts with and stabilizes c-Jun

**Fig. 5:**
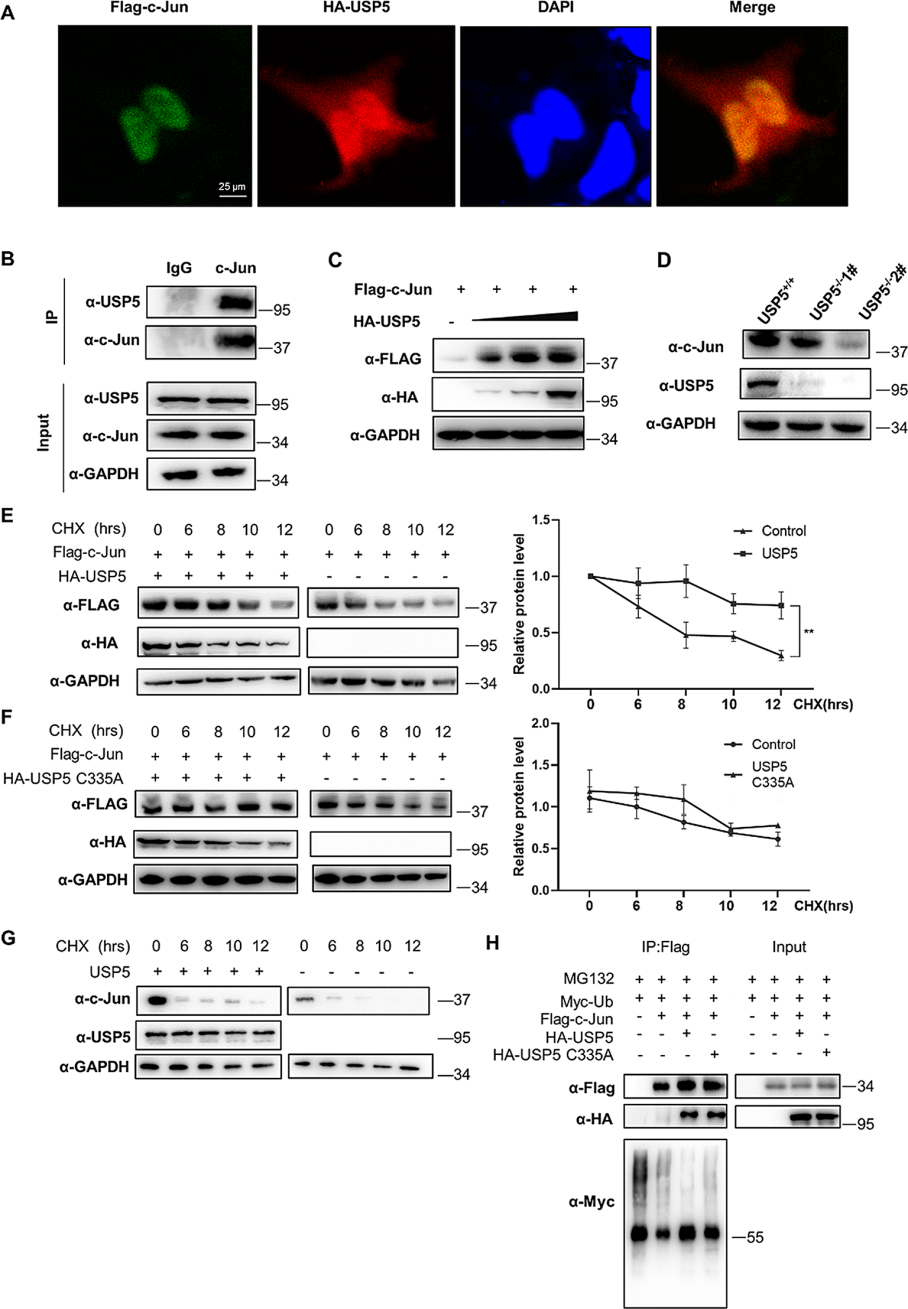
USP5 interacts with c-Jun and promotes c-Jun protein stability. **A** Immunofluorescence images of HA-USP5 (red) and FLAG-c-Jun (green) in HEK293T cells. DAPI was used as a nuclear stain (blue). **B** Immunoprecipitation experiments showed the endogenous interaction between USP5 and c-Jun in T24 cells. **C** Cells were transfected with increasing amounts of the HA-USP5 (0, 100, 200, 500 ng) and FLAG-c-Jun plasmids, and western blotting was performed to determine the effect of USP5 protein levels on c-Jun expression in HEK293T cells. **D** Western blot analysis of c-Jun expression in USP5-deficient T24 cells. **E** HEK293T cells were transfected with FLAG-c-Jun with or without HA-USP5 as indicated. Western blot analysis of c-Jun stability after treatment with CHX (50 µg/mL) for the indicated time. GAPDH served as a control. **F** HEK293T cells were transfected with FLAG-c-Jun with or without HA-USP5 C335A as indicated. Western blot analysis of c-Jun stability after treatment with CHX (50 µg/mL) for the indicated times. GAPDH served as a control. **G** Western blot analysis of c-Jun stability in USP5-deficient cells after treatment with CHX (50 µg/mL) for the indicated time. GAPDH served as a control. **H** HEK293T cells were cotransfected with FLAG-c-Jun and Myc-Ub with or without HA-USP5 or HA-USP5 C335A, treated with MG132 (10 µM) for 6 h and then subjected to ubiquitination assays. The data are representative of 3 independent experiments. Statistical significance was analysed by ANOVA or Student’s t test. **P < 0.01

We next investigated the relationship between USP5 and the JNK pathway. We examined the interaction of USP5 with several key proteins (Fig. S2). Immunofluorescence staining showed that USP5 and c-Jun were localized in the nucleus upon plasmid-mediated exogenous overexpression in HEK293T cells (Fig. 5A). Then, coimmunoprecipitation was used to indicate that USP5 interacts with c-Jun in T24 cells (Fig. 5B). We investigated whether USP5 affects the stability of c-Jun. The figure shows that c-Jun protein levels were increased when USP5 was overexpressed and that this increase occurred in a dose-dependent manner (Fig. 5C). Figure 5D shows that USP5 deficiency reduced c-Jun levels. To establish the effect of USP5 on c-Jun stability, we performed cycloheximide (CHX) chase assays to verify the time course of c-Jun degradation. The half-life of c-Jun increased after transfection of the USP5 plasmid (Fig. 5E), while the half-life of c-Jun was not increased by transfection of the USP5 C335A plasmid (Fig. 5F). The half-life of c-Jun was significantly reduced in USP5-deficient cells (Fig. 5G). Considering the function of USP5 as a DUB, we next investigated whether USP5 can reduce the ubiquitination of c-Jun. The results showed that the levels of ubiquitinated c-Jun were reduced by overexpression of WT USP5 compared with those in control cells. However, overexpression of USP5 C335A abolished this reduction (Fig. 5H).

In summary, these results show that USP5 interacts with c-Jun and regulates its stability by inhibiting its ubiquitination.

### USP5 deficiency inhibits tumour formation in vivo

**Fig. 6:**
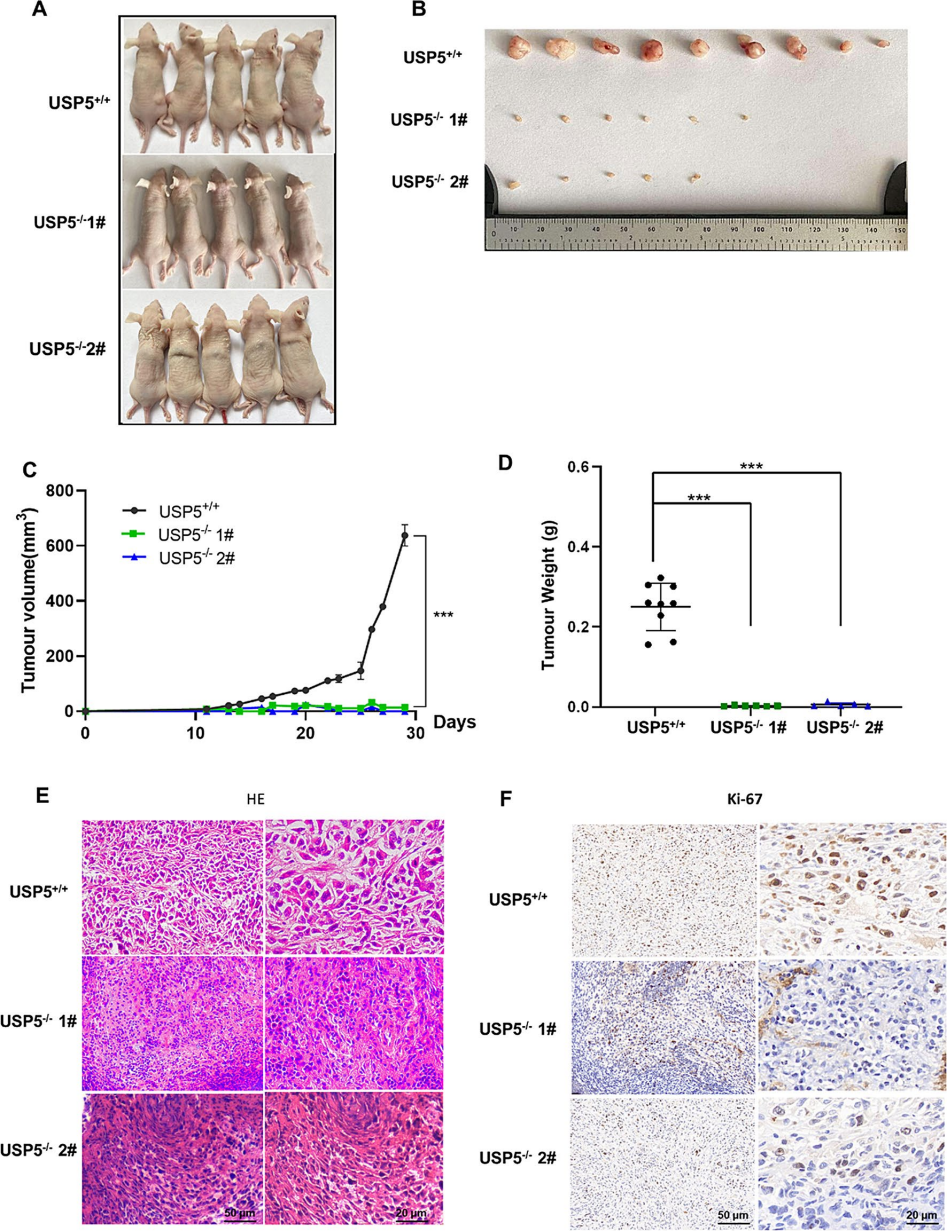
USP5 deficiency represses tumour formation in vivo. **A**, **B** Tumours were harvested from euthanized mice and weighed, and representative images are shown. **C** Tumours were measured every 2 days, and the tumour volume was plotted. **D** Quantitative results of tumour weight. **E** Tumour tissues from each group of nude mice were prepared as paraffin sections and stained with HE. **F** Tumour tissues from each group of nude mice were prepared as paraffin sections and stained with an anti-Ki67 antibody. Statistical significance was analysed by ANOVA or Student’s t test. ***P < 0.001

To investigate whether USP5 influences tumorigenesis in vivo, WT and USP5-knockout T24 cells were injected into the flanks of nude mice. As shown in Fig. 6A–C, the tumour volume in nude mice injected with USP5-knockout cells was significantly smaller than that in nude mice injected with the control T24 cells, and the tumours in the USP5-knockout group grew markedly slower than those in the WT T24 cell-injected group (Fig. 6D). The tumours were sectioned and stained with HE and an anti-Ki67 antibody (Fig. 6E, F). The results revealed that depletion of USP5 inhibited the activity of T24 cells in vivo. Altogether, these results indicate that USP5 potentially promotes bladder cancer growth in vivo.

## Discussion

DUBs are essential for maintaining ubiquitin homeostasis and are required for diverse cellular functions. There are more than 100 DUBs encoded in the human genome. USP5, also called ubiquitin isopeptidase (ISOT), has been reported to be involved in multiple cellular processes, including stress responses, DNA repair and inflammatory responses [30]. USP5 has also been found to be associated with cancers, including breast, prostate, testicular and urothelial cancers. Several studies have revealed the role of USP5 in HCC, PDAC and CRC. However, the mechanism of USP5 in bladder cancer needs to be determined. First, we analysed the expression of USP5 in bladder cancer in the online GEPIA database, and the results suggested that USP5 is upregulated in bladder cancer patients. The results of IHC staining experiments with a tissue microarray were revealed that USP5 was highly expressed in bladder cancer compared with normal tissue. To determine the function of USP5 in bladder cancer, we constructed USP5-overexpressing and USP5-deficient cancer cell lines. The phenotype results suggested that USP5 promotes the development and progression of bladder cancer. Next, we performed RNA-seq analysis and luciferase pathway screening to explore the mechanism of USP5 in bladder cancer. The results pointed to the involvement of the JNK pathway. Previous studies have revealed that the JNK pathway is strongly associated with bladder cancer [31]. To explore how USP5 activates the JNK signalling pathway, we performed coimmunoprecipitation experiments to determine whether USP5 can interact with important molecules in the JNK signalling pathway. We verified the physical association between USP5 and c-Jun. We detected USP5 and c-Jun colocalization in the nucleus.

Posttranslational modifications, including phosphorylation, ubiquitination, and acetylation, are important to the function of c-Jun. Ubiquitination is responsible primarily posttranslational modification for the stability of c-Jun. Several studies have revealed that c-Jun is ubiquitinated by several E3 ubiquitin ligases [20, 32]. In 2018, Lin et al. published a paper in which they described that USP6 regulates the stability of the c-Jun protein [33]. We sought to determine whether USP5 could regulate the stability of the c-Jun protein. We further verified that overexpression of USP5 could stabilize the c-Jun protein. It has been reported that USP5 cleaves K6-linked, K29-linked, K48-linked, K63-linked and linear ubiquitin chains, especially Lys48-linked polyubiquitin chains [34]. To determine whether USP5 stabilizes the c-Jun protein by deubiquitination, we constructed a plasmid expressing the catalytically inactive mutant USP5 C335A. CHX chase assays and deubiquitination assays were performed, and the results suggested that USP5 stabilizes the c-Jun protein by inhibiting its ubiquitination. Considering the phosphorylation of c-Jun is closely related to its stability, further studies need to be performed to evaluate whether USP5 affects c-Jun phosphorylation. Finally, an in vivo xenograft mouse model was used to study the role of USP5 in bladder cancer. The results demonstrated that USP5 potentially promotes bladder tumour growth.

USPs involves in a wide range of pathological processes of malignancy, they have been considered targets for drug development. The development of USP inhibitors has also become a perspective and possibility for cancer therapy. Several USP5 inhibitors have been developed to treat human cancers. Such as WP1130 [35], PYR-41 [36] formononetin [24]. However, there was few findings suggest that USP5 could be a potential target for bladder cancer therapy. Our experimental results showed that USP5 is associated with poor prognosis in bladder cancer, Further studies need to be performed to evaluate whether this treatment strategy works against bladder cancer development and progression through specific inhibitors selectively targeting USP5.

In conclusion, our experimental results showed that USP5 is associated with poor prognosis in bladder cancer. USP5 plays an oncogenic role through deubiquitination of c-Jun, which is an important downstream target of the JNK pathway in bladder cancer. Our study reveals a new potential therapeutic target for bladder cancer.

### Electronic supplementary material

Below is the link to the electronic supplementary material.

**Supplementary Material 1**: **Fig. S1** Expression of USP5 in bladder cancer cell lines. **Fig. S2** Identification of the interaction between USP5 and key molecules of JNK signaling pathway

## Data Availability

The experimental data sets and materials generated and/or analyzed during the current study are available from the corresponding author upon reasonable request. Raw sequences were submitted to the National Center for Biotechnology Information under Bioproject accession number PRJNA1007642.

## Abbreviations

BC: Bladder cancer
CCK8: Cell Counting Kit-8
CHX: Cycloheximide
CRC: colorectal cancer
DUB: deubiquitinating enzyme
HCC: hepatocellular carcinoma
IHC: immunohistochemistry
JNK: c-Jun N-terminal kinase
MIBC: muscle-invasive bladder cancer
NMIBC: non-muscle-invasive bladder cancer
PDAC: pancreatic ductal adenocarcinoma
RNA-seq: RNA sequencing
USP: ubiquitin-specific protease
