# Supplementary material for: USP5 facilitates bladder cancer progression by stabilizing the c-Jun protein
Source: Cancer Cell Int. 2024 Jan 16;24:32. doi: 10.1186/s12935-024-03222-7 (PMC10792936; doi:10.1186/s12935-024-03222-7)
Supplement: Supplementary file 1 — Supplementary Material 1: Fig. S1 Expression of USP5 in bladder cancer cell lines. Fig. S2 Identification of the interaction between USP5 and key molecules of JNK signaling pathway [file 12935_2024_3222_MOESM1_ESM.docx]

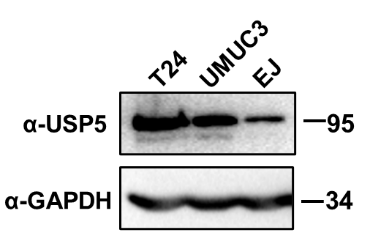


Fig S1 USP5 expression levels in bladder cancer cell lines


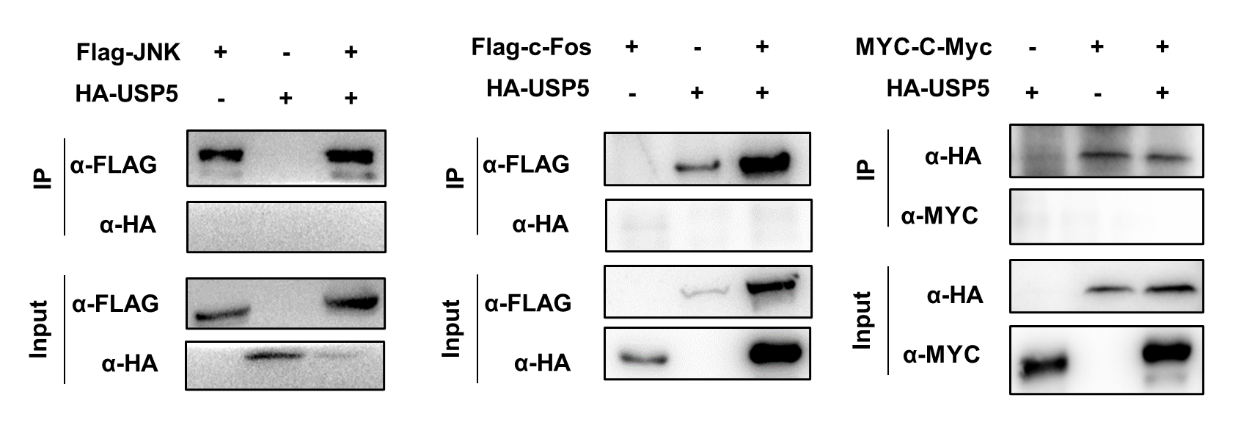


Fig S2 Immunoprecipitation experiments showed there is no interaction between USP5 and JNK, c-FOS, C-myc.
